# Computational pharmacogenotype extraction from clinical next-generation sequencing

**DOI:** 10.3389/fonc.2023.1199741

**Published:** 2023-07-04

**Authors:** Tyler Shugg, Reynold C. Ly, Wilberforce Osei, Elizabeth J. Rowe, Caitlin A. Granfield, Ty C. Lynnes, Elizabeth B. Medeiros, Jennelle C. Hodge, Amy M. Breman, Bryan P. Schneider, S. Cenk Sahinalp, Ibrahim Numanagić, Benjamin A. Salisbury, Steven M. Bray, Ryan Ratcliff, Todd C. Skaar

**Affiliations:** ^1^ Division of Clinical Pharmacology, Department of Medicine, Indiana University School of Medicine, Indianapolis, IN, United States; ^2^ Division of Diagnostic Genetics and Genomics, Department of Medical and Molecular Genetics, Indiana University School of Medicine, Indianapolis, IN, United States; ^3^ Division of Hematology/Oncology, Department of Medicine, Indiana University School of Medicine, Indianapolis, IN, United States; ^4^ Center for Cancer Research, National Cancer Institute, National Institute of Health, Bethesda, MD, United States; ^5^ Department of Computer Science, University of Victoria, Victoria, BC, Canada; ^6^ LifeOmic Inc., Indianapolis, IN, United States

**Keywords:** pharmacogenetic algorithm, next-generating sequencing, whole genome sequencing (WGS), whole exome sequencing (WES), pharmacogenomics (PGx), pharmacogenetics (PGx)

## Abstract

**Background:**

Next-generation sequencing (NGS), including whole genome sequencing (WGS) and whole exome sequencing (WES), is increasingly being used for clinic care. While NGS data have the potential to be repurposed to support clinical pharmacogenomics (PGx), current computational approaches have not been widely validated using clinical data. In this study, we assessed the accuracy of the Aldy computational method to extract PGx genotypes from WGS and WES data for 14 and 13 major pharmacogenes, respectively.

**Methods:**

Germline DNA was isolated from whole blood samples collected for 264 patients seen at our institutional molecular solid tumor board. DNA was used for panel-based genotyping within our institutional Clinical Laboratory Improvement Amendments- (CLIA-) certified PGx laboratory. DNA was also sent to other CLIA-certified commercial laboratories for clinical WGS or WES. Aldy v3.3 and v4.4 were used to extract PGx genotypes from these NGS data, and results were compared to the panel-based genotyping reference standard that contained 45 star allele-defining variants within *CYP2B6*, *CYP2C8*, *CYP2C9*, *CYP2C19*, *CYP2D6*, *CYP3A4*, *CYP3A5*, *CYP4F2*, *DPYD*, *G6PD*, *NUDT15*, *SLCO1B1*, *TPMT*, and *VKORC1*.

**Results:**

Mean WGS read depth was >30x for all variant regions except for *G6PD* (average read depth was 29 reads), and mean WES read depth was >30x for all variant regions. For 94 patients with WGS, Aldy v3.3 diplotype calls were concordant with those from the genotyping reference standard in 99.5% of cases when excluding diplotypes with additional major star alleles not tested by targeted genotyping, ambiguous phasing, and *CYP2D6* hybrid alleles. Aldy v3.3 identified 15 additional clinically actionable star alleles not covered by genotyping within *CYP2B6*, *CYP2C19*, *DPYD*, *SLCO1B1*, and *NUDT15*. Within the WGS cohort, Aldy v4.4 diplotype calls were concordant with those from genotyping in 99.7% of cases. When excluding patients with *CYP2D6* copy number variation, all Aldy v4.4 diplotype calls except for one *CYP3A4* diplotype call were concordant with genotyping for 161 patients in the WES cohort.

**Conclusion:**

Aldy v3.3 and v4.4 called diplotypes for major pharmacogenes from clinical WES and WGS data with >99% accuracy. These findings support the use of Aldy to repurpose clinical NGS data to inform clinical PGx.

## Introduction

Short-read next-generation sequencing (NGS) has emerged as a valuable tool to support clinical diagnosis and decision making in a variety of therapeutic disciplines (1–4). Clinical NGS frequently utilizes whole genome approaches (i.e., whole exome sequencing [WES] or whole genome sequencing [WGS]) that sequence gene regions across the genome. As a result, clinical NGS data contain a wealth of genetic information with relevance for pharmacogenetics (PGx), the practice of using genetic information to guide safe and effective medication use (5, 6).

Recently, computational methods have been developed to extract PGx-relevant genotypes, including star alleles for cytochrome P450 (CYP) enzymes, from NGS data without requiring additional genetic testing (7–13). These methods have primarily been validated using genetic reference material (e.g., cell lines from the Genetic Testing Materials Coordination Program [GeT-RM]) (14–16); however, in order to be suitable for clinical implementation, it is critical to verify the accuracy of computational genotype extraction methods using clinical NGS data derived from patient samples. Clinical NGS data can present numerous challenges that limit precise genotype determination, including low read depth, incomplete coverage of PGx-relevant loci, the inability to phase variants, and difficulty resolving large-scale structural variations (17). Computational genotyping methods have been iteratively developed towards the goal of overcoming these challenges, but their ability to accurately extract genotypes from clinical NGS data remains largely unestablished.

We previously assessed the performance of the computational genotyping software Aldy (version 3.3) using clinical WES (17). We found that Aldy v3.3 genotype calls were concordant with those from our validated genotyping reference standard for 13 major pharmacogenes; however, we noted important limitations of Aldy v3.3 that included the inability to determine *CYP2D6* copy number or to phase clinically relevant, multi-variant alleles in *CYP2B6*, *CYP2D6*, and *TPMT*. To date, we are not aware of any study assessing the accuracy of computational methods for extracting pharmacogenotypes from clinical WGS data. Therefore, the primary objective of this study was to assess the accuracy of Aldy v.3.3 calls for 14 major pharmacogenes using WGS files from patients (n=100) seen at our institutional molecular solid tumor board. Since a new Aldy version (v4.4) was recently released to support improved copy number calling, variant calling, and phasing (18), we also assessed the performance of Aldy v4.4 on the WGS cohort and on the patient cohort (n=161) from our past WES validation.

## Materials and methods

### Patient enrollment

Written informed consent to participate in the study was obtained from patients with solid tumors treated at the Indiana University Precision Genomics Clinic (Indianapolis, IN) from September 2017 to March 2020. Patients consented to provide whole blood samples for genomic analyses, which consisted of (1) pharmacogenetics genotyping performed at the Indiana University Pharmacogenomics Laboratory and (2) WGS performed at NantOmics or WES performed at Ashion Analytics. The study enrolled a total of 100 patients with WGS and 164 patients with WES. The study protocol and the parent Indiana University Total Cancer Care Protocol were approved by the Indiana University Institutional Review Board. Patient demographic and clinical data, including age, sex, race/ethnicity, and date and type of first cancer diagnosis, were obtained from a Total Cancer Care Protocol study database.

### Panel-based genotyping and orthogonal confirmation of additional variants identified by Aldy

We have previously described our panel-based genotyping method and *CYP2D6* copy number testing in detail (17, 19). To summarize, genotyping was performed at the Clinical Laboratory Improvement Amendments (CLIA)-certified, College of American Pathologists (CAP)-accredited Indiana University Pharmacogenomics Laboratory using a validated, custom-designed OpenArray^®^ platform (ThermoFisher, Waltham, MA). The genes included on the genotyping platform were *CYP2B6*, *CYP2C8*, *CYP2C9*, *CYP2C19*, *CYP2D6* (including copy number), *CYP3A4*, *CYP3A5*, *CYP4F2*, *DPYD*, *G6PD*, *SLCO1B1*, *TPMT*, and *VKORC1*. The complete list of the 45 variants on the genotyping platform are shown in **Supplemental Table 1**. *NUDT15* was added to the genotyping platform after genotyping was completed for the WGS cohort, necessitating orthogonal confirmation for any *NUDT15* variant called by Aldy in the WGS cohort. Patients whose DNA sample passed clinical laboratory quality control (all 161 patients in the WES cohort and 94/100 patients in the WGS cohort) were genotyped.

Orthogonal confirmation was performed for any variant in the 14 assessed pharmacogenes that was detected by Aldy, not included in the genotyping platform, and had actionable recommendations in current Clinical Pharmacogenetics Implementation Consortium (CPIC) guidelines. Detailed descriptions of these orthogonal confirmation methods have been previously published (17). To summarize, these variants were confirmed with PCR-based genotyping using commercial TaqMan reagents, when available, and a positive genomic control from a Coriell cell line with known genotypes from 1000 Genomes for TaqMan allelic discrimination. All other variants were confirmed using Sanger sequencing methods, with primers for each variant being designed using the National Center for Biotechnology Information Primer-Blast tool (https://www.ncbi.nlm.nih.gov/tools/primer-blast) and synthesized by Integrated DNA Technologies, Inc. The assay identification numbers of the TaqMan reagents and the sequences of the Sanger sequencing primers are provided in **Supplemental Table 2**.

### Next-generation sequencing

Whole blood samples were sent to the following CLIA-certified, CAP-accredited clinical laboratories for next-generation sequencing: NantOmics Laboratory (Culver City, CA) for WGS or Ashion Analytics Laboratory (Phoenix, AZ) for WES. Germline NGS was performed as part of each laboratory’s commercial paired normal/tumor sequencing workflow, available as GPS Cancer^™^ from NantOmics and as Genomic Enabled Medicine Exome Testing (GEM ExTra) from Ashion Analytics. NGS methods for GPS Cancer^™^ and GEM ExTra have been previously published (20, 21). Briefly, germline DNA was isolated from whole blood samples, and DNA sequencing libraries were prepared using a custom xGEN target capture kit (GEM ExTra) or a KAPA Hyper prep kit (GPS Cancer^™^). Libraries were sequenced using Illumina sequencing platforms, and sequencing data were analyzed using proprietary bioinformatics workflows. Sequencing reads were aligned to the Genome Reference Consortium Human Build 37 (GRCh37)/Human Genome version 19 (hg19). Binary alignment map (BAM) and variant calling files (VCFs) were returned from each laboratory and transferred into the Precision Health Cloud (LifeOmic, Indianapolis, IN) for analysis.

### Analysis of next-generation sequencing data with Aldy and Cyrius

The Aldy and Cyrius genotype extraction methods have been described in detail in previous publications (9, 13, 18). Aldy v3.3 and v4.4 were downloaded via pypi packages (links available on the Aldy Github: https://github.com/0xTCG/aldy), and the Cyrius v1.1.1 source code was downloaded from the Cyrius Github (https://github.com/Illumina/Cyrius/releases/tag/v1.1.1). All callers were operationalized in the LifeOmic Precision Health Cloud using JupyterLab notebooks. Read depth was analyzed from NantOmics WGS BAM files for all variants included in the genotyping platform using Samtools and outputted in the.csv file format for analysis. The same method was previously used to analyze read depth from Ashion WES BAM files (17). Aldy performed genotype extraction for the major pharmacogenes contained on the genotyping platform, which included 14 genes from WGS BAM files and 13 genes from WES BAM files (*VKORC1* was not assessed using WES BAMs since the rs9923231 promoter variant was not covered). The “WXS” and “Illumina” sequencing profiles were used for Aldy genotype extraction from WES data and WGS data, respectively. All other default Aldy parameters were used. Aldy output files, which were outputted in the.txt file format for analysis, included the list of variants detected and the assembled diplotype call. Cyrius was used to perform genotype extraction for the four BAM files that had discordant *CYP2D6* genotype calls with Aldy v3.3 or v4.4 (or both) relative to the genotyping reference standard.

### Analytical assessment of Aldy

The accuracy of Aldy calls was assessed at the variant and diplotype levels via comparison to clinical laboratory genotype results or results from orthogonal methods. Diplotypes called by Aldy with ambiguous phasing or with hybrid alleles calls containing *CYP2D6* and *CYP2D7* (herein referred to as “*CYP2D6* hybrid alleles”) were excluded from the diplotype concordance analysis. Standard analytical parameters for diagnostic testing, including analytical sensitivity and specificity, intra- and inter-assay concordance, and overall accuracy, were assessed for each PGx-relevant variant in our cohort possessed by ≥1 study patient. Additional star alleles identified by Aldy were deemed “clinically actionable” if they had clinical function annotations other than “normal function” by the Pharmacogene Variation Consortium (PharmVar) and PharmGKB (22, 23).

### Determination of clinical impact of additional star alleles identified by Aldy

For patients with additional clinically actionable star alleles identified by Aldy (that were able to be unambiguously phased), medication data was obtained since each patient’s respective date of first cancer diagnosis via query of the Indiana Health Information Exchange, a statewide electronic health record data repository, as previously described (19). Patient pharmacogene phenotypes were categorized based on current diplotype to phenotype translation tables curated by PharmGKB and CPIC (22). Patient medications were considered actionable if current CPIC guidelines provide “strong” or “moderate” recommendations to alter therapy with the medication (i.e., prescribe an alternative medication, adjust the dose, increase monitoring, or perform additional functional testing) based on each patient’s Aldy-predicted phenotype (relative to their phenotype as predicted by the genotyping reference standard) (24). Medications that were considered actionable for each patient are summarized in **Supplemental Table 3**.

## Results

### Patient population

For comparison to the genotyping reference standard, Aldy calls were extracted from clinical NGS data in a cohort of 264 patients, which consisted of 100 patients with clinical WGS and 164 patients with clinical WES. The demographic and clinical characteristics of the overall study population are presented in **Table 1**, with characteristics for the WGS cohort provided in **Supplemental Table 4**. Demographic and clinical characteristics for the WES cohort have been previously published (17). Within the overall study population, the median age at first cancer diagnosis was 56 years old (interquartile range: 16). Our population was 53% male, 85% white race, and 99% non-Hispanic ethnicity. The most frequent primary cancer diagnoses in our population were breast (15%), colorectal (12%), pancreatic (11%), prostate (10%), and soft tissue sarcoma (7%).

**Table 1:** Demographic and clinical characteristic of study population.

Variable	Full Study Population (n=264)
Age in years at first cancer diagnosis (Median [IQR])	56 (16)
Sex (Count [Percent])	
Male	139 (52.7%)
Female	125 (47.3%)
Race (Count [Percent])	
White	225 (85.2%)
Black	26 (9.8%)
Unknown	10 (3.8%)
Asian	3 (1.1%)
Ethnicity (Count [Percent])	
Non-Hispanic	262 (99.2%)
Hispanic	2 (0.8%)
Primary Cancer type (Count [Percent])	
Breast	39 (14.8%)
Colorectal	32 (12.1%)
Pancreatic	30 (11.4%)
Prostate	27 (10.2%)
Soft tissue sarcoma	18 (6.8%)
Ovarian	11 (4.2%)
Bladder	9 (3.4%)
Esophageal	9 (3.4%)
Cholangiocarcinoma	8 (3.0%)
Head and neck	8 (3.0%)
Non-small cell lung	8 (3.0%)
Glioblastoma	7 (2.7%)
Melanoma	5 (1.9%)
Renal	5 (1.9%)
Other*	48 (18.2%)

*The “other” category consisted of all primary cancer types which occurred in fewer than 5 patients in our study population.

### Coverage of pharmacogenetic variants by next-generation sequencing

Read depth analysis was performed to confirm that the chromosomal positions for the 45 variants included on our genotyping panel were sufficiently covered within our NGS data. The average read depth at each position for the WGS cohort is shown in **Supplemental Table 5**, and the average read depth for the WES cohort has been previously published (17). Within the WGS cohort, all PGx-relevant loci had an average read depth above the minimum recommended WGS coverage threshold of 30 reads except for the *G6PD* “A” and c.202G>A “A-” alleles, which had an average read depth of 29 ± 15 (mean ± standard deviation) reads (25). Within the WES cohort, all PGx loci had an average read depth above 100 reads except for CYP3A4*22, which had a read depth of 44 ± 12 reads. These findings confirmed that all PGx loci were adequately covered in the NGS data to proceed with the analytical assessment of the Aldy method.

### Analytical assessment *of Aldy* v3.3 using whole genome sequencing

An analysis validating Aldy v3.3 genotyping calls within the WES cohort has been previously published (17). For 94 patients in the WGS cohort, Aldy v3.3 calls were compared to the genotyping reference standard at both the variant and diplotype levels, with results shown in **Tables 2**, **3**, respectively. Aldy v3.3 calls were concordant with the reference standard for all assessed variants within *CYP2C8*, *CYP2C9*, *CYP2C19*, *CYP3A5*, *CYP4F2*, *DPYD*, *G6PD*, *NUDT15*, *SLCO1B1*, *TPMT*, and *VKORC1*, as reflected by point estimates of 100% for analytical sensitivity, analytical specificity, and overall accuracy. The following discordant Aldy v3.3 calls were observed: one CYP2B6*6 (c.516G>T) call; two CYP2D6*2 (c.886C>T) calls; two CYP2D6*2 (c.1457G>C) calls; and one CYP3A4*22 (c.522-191C>T) call. Interrogation of the WGS data revealed that five of these heterozygous variants had variant allele frequencies just outside of the 25-75% threshold used by Aldy v3.3 to classify variants as heterozygous in diploid genotype calls, which explains the discordant results. Aldy v3.3’s intra- and inter-assay concordance, defined as the ability to reproduce the same call for the same sequencing file within the same and subsequent runs, respectively, were confirmed to be 100% for all assessed variants. Stemming from the variant level results, five diplotype calls (WGS16 in *CYP2B6*, WGS5, WGS35, and WGS55 in *CYP2D6*, and WGS73 in *CYP3A4*) were discordant. Aldy v3.3 identified additional alleles not covered by the genotyping reference standard for 196 diplotype calls, which included 15 clinically actionable alleles within *CYP2B6* (1), *CYP2C19* (3), *DPYD* (8), *SLCO1B1* (2), and *NUDT15* (1); these additional clinically actionable alleles were confirmed by orthogonal genotyping methods. All other 1,005 diplotype calls were concordant across the 14 assessed genes.

**Table 2:** Variant-level assessment of Aldy v3.3 genotype extraction from clinical WGS.

Variant	Variant Allele Count in WGS Cohort (Total Chromosomes Tested)	Analytical Parameters Assessed in WGS Cohort				
		Analytical Sensitivity [% (95% CI)]	Analytical Specificity [% (95% CI)]	Inter-Assay Concordance (%)	Intra-Assay Concordance (%)	Overall Accuracy (%)
CYP2B6*6 (c.516G>T)	64 (188)	98.4% (92-100)	100% (97-100)	100%	100%	99.5%
CYP2B6*8 (c.415A>G)	1 (1)	100% (21-100)	N/A^*^	100%	100%	100%
CYP2B6*18 (c.983T>C)	1 (188)	100% (21-100)	100% (98-100)	100%	100%	100%
CYP2C8*2 (c.805A>T)	4 (188)	100% (51-100)	100% (98-100)	100%	100%	100%
CYP2C8*3 (c.416G>A)	20 (188)	100% (84-100)	100% (98-100)	100%	100%	100%
CYP2C8*4 (c.792C>G)	6 (188)	100% (61-100)	100% (98-100)	100%	100%	100%
CYP2C9*2 (c.430C>T)	21 (188)	100% (85-100)	100% (98-100)	100%	100%	100%
CYP2C9*3 (c.1075A>C)	5 (188)	100% (57-100)	100% (98-100)	100%	100%	100%
CYP2C9*5 (c.1080C>G)	0 (188)	N/A	100% (98-100)	100%	100%	100%
CYP2C9*6 (c.818delA)	1 (188)	100% (21-100)	100% (98-100)	100%	100%	100%
CYP2C9*8 (c.449G>A)	0 (188)	N/A	100% (98-100)	100%	100%	100%
CYP2C9*11 (c.1003C>T)	2 (188)	100% (34-100)	100% (98-100)	100%	100%	100%
CYP2C19*2 (c.681G>A)	24 (188)	100% (86-100)	100% (98-100)	100%	100%	100%
CYP2C19*3 (c.636G>A)	0 (188)	N/A	100% (98-100)	100%	100%	100%
CYP2C19*4 (c.1A>G)	0 (188)	N/A	100% (98-100)	100%	100%	100%
CYP2C19*6 (c.395G>A)	0 (188)	N/A	100% (98-100)	100%	100%	100%
CYP2C19*8 (c.358T>C)	1 (188)	100% (21-100)	100% (98-100)	100%	100%	100%
CYP2C19*9 (c.431G>A)	1 (1)	100% (21-100)	N/A^*^	100%	100%	100%
CYP2C19*10 (c.680C>T)	0 (188)	N/A	100% (98-100)	100%	100%	100%
CYP2C19*17 (g.-806C>T)	43 (188)	100% (92-100)	100% (97-100)	100%	100%	100%
CYP2C19*35 (c.332-23A>G)	2 (2)	100% (34-100)	N/A^*^	100%	100%	100%
CYP2D6*2 (c.886C>T)	65 (188)	98.5% (92-100)	99.2% (96-100)	100%	100%	98.9%
CYP2D6*2 (c.1457G>C)	94 (188)	97.9% (93-99)	100% (96-100)	100%	100%	98.9%
CYP2D6*3 (c.775del)	4 (188)	100% (51-100)	100% (98-100)	100%	100%	100%
CYP2D6*4 (c.506-1G>A)	27 (188)	100% (88-100)	100% (98-100)	100%	100%	100%
CYP2D6*6 (c.454del)	1 (188)	100% (21-100)	100% (98-100)	100%	100%	100%
CYP2D6*7 (c.971A>C)	0 (188)	N/A	100% (98-100)	100%	100%	100%
CYP2D6*8 (c.505G>T)	0 (188)	N/A	100% (98-100)	100%	100%	100%
CYP2D6*9 (c.841_843del)	3 (188)	100% (44-100)	100% (98-100)	100%	100%	100%
CYP2D6*10 (c.100C>T)	29 (188)	100% (88-100)	100% (98-100)	100%	100%	100%
CYP2D6*14 (c.505G>A)	0 (200)	N/A	100% (98-100)	100%	100%	100%
CYP2D6*17 (c.320C>T)	3 (188)	100% (44-100)	100% (98-100)	100%	100%	100%
CYP2D6*29 (c.1012G>A)	1 (188)	100% (21-100)	100% (98-100)	100%	100%	100%
CYP2D6*41 (c.985 + 39G>A)	21 (188)	100% (85-100)	100% (98-100)	100%	100%	100%
CYP3A4*2 (c.664T>C)	0 (188)	N/A	100% (98-100)	100%	100%	100%
CYP3A4*22 (c.522-191C>T)	14 (188)	92.9% (69-99)	100% (98-100)	100%	100%	99.5%
CYP3A5*3 (c.219-237A>G)	160 (188)	100% (98-100)	100% (88-100)	100%	100%	100%
CYP3A5*6 (c.454del)	0 (188)	N/A	100% (98-100)	100%	100%	100%
CYP3A5*7 (c.1035dup)	3 (188)	100% (44-100)	100% (98-100)	100%	100%	100%
CYP4F2*3 (c.1297G>A)	48 (188)	100% (93-100)	100% (97-100)	100%	100%	100%
DPYD*2 (c.1905 + 1G>A)	1 (188)	100% (21-100)	100% (98-100)	100%	100%	100%
DPYD*7 (c.299delTCAT)	1 (1)	100% (21-100)	N/A^*^	100%	100%	100%
*DPYD* D949V (c.2846A>T)	2 (2)	100% (34-100)	N/A^*^	100%	100%	100%
*DPYD* HapB3 (c.1129-5923C>G)	4 (4)	100% (51-100)	N/A^*^	100%	100%	100%
*DPYD* Y186C (c.557A>G)	1 (1)	100% (21-100)	N/A^*^	100%	100%	100%
*G6PD* A- (c.202G>A)	1 (188)	100% (21-100)	100% (98-100)	100%	100%	100%
*G6PD* A (c.376A>G)	5 (188)	100% (57-100)	100% (98-100)	100%	100%	100%
NUDT15*9 (c.38GAGTCG[2])	1 (1)	100% (21-100)	N/A^*^	100%	100%	100%
SLCO1B1*5 (c.521T>C)	19 (188)	100% (83-100)	100% (98-100)	100%	100%	100%
SLCO1B1*9 (c.1463G>C)	2 (2)	100% (34-100)	N/A^*^	100%	100%	100%
TPMT*2 (c.238G>C)	0 (188)	N/A	100% (98-100)	100%	100%	100%
TPMT*3 (c.460G>A)	4 (188)	100% (51-100)	100% (98-100)	100%	100%	100%
TPMT*3 (c.719A>G)	4 (188)	100% (51-100)	100% (98-100)	100%	100%	100%
*VKORC1* (c.-1639G>A)	56 (188)	100% (94-100)	100% (97-100)	100%	100%	100%

*Not applicable (N/A): Alleles that were not covered by the genotyping platform and required orthogonal confirmation were only assessed in individuals predicted by Aldy to be carriers of the variant. As a result, analytical specificity was not assessed for these variants.

**Table 3:** Diplotype-level assessment of Aldy v3.3 genotype extraction from clinical WGS.

Gene	Concordant/Equivalent diplotypes^1^	Discordant Diplotypes	Detection of additional major star alleles^2,3^	Indeterminate diplotypes^4^	*CYP2D6* Hybrid diplotype calls^4^	Total diplotypes compared
*CYP2B6*	63	1	29 (1)	(1)	N/A*	**93**
*CYP2C8*	94	0	0	0	N/A	**94**
*CYP2C9*	93	0	1	0	N/A	**94**
*CYP2C19*	89	0	5 (3)	0	N/A	**94**
*CYP2D6*	58	3	21	0	(12)	**83**
*CYP3A4*	90	1	3	0	N/A	**94**
*CYP3A5*	94	0	0	0	N/A	**94**
*CYP4F2*	68	0	26	0	N/A	**94**
*DPYD*	21	0	73 (8)	0	N/A	**94**
*G6PD*	94	0	0	0	N/A	**94**
*NUDT15*	0	0	1 (1)	0	N/A	**1**
*SLCO1B1*	60	0	34 (2)	0	N/A	**94**
*TPMT*	87	0	3	(4)	N/A	**90**
*VKORC1*	94	0	0	0	N/A	**94**
**Total**	**1005**	**5**	**196 (15)**	**(5)**	**(12)**	**1207**

1.Alleles with the same tag variant(s) (e.g., CYP2D6*2 versus CYP2D6*34 and CYP2D6*39) were considered as equivalent alleles.

2.Additional clinically actionable star alleles were defined as alleles classified as having increased function, decreased function, or no function within current Clinical Pharmacogenetics Implementation Consortium (CPIC) guidelines that were not included on our genotyping reference standard. Since no current CPIC guidelines include *CYP2C8* or *CYP3A4* genotype-guided recommendations, there was no potential to identify additional clinically actionable star alleles. Diplotypes containing additional clinically actionable star alleles are in parentheses.

3.*DPYD* no longer uses star allele nomenclature according to PharmVar, so any additional variant called by Aldy for *DPYD* was interpreted as an additional allele.

4.Values in parentheses were not included in diplotype concordance analysis for subjects with indeterminate diplotypes or *CYP2D6* hybrid calls.

*Not applicable (N/A): Hybrid diplotype calls are only relevant for *CYP2D6*.

### Analytical assessment of *Aldy v4.4* using whole exome and whole genome sequencing

Aldy v4.4 diplotype calls were extracted from WES and WGS sequencing files. For three of the WES files, Aldy v4.4 returned a warning that “the average coverage was too low” and did not produce output. **Table 4** summarizes Aldy v4.4 diplotype calls compared to the genotyping reference standard for the remaining 161 patients in the WES cohort across the 13 assessed pharmacogenes. With the exception of 23 *CYP2D6* diplotypes that were excluded from analysis due to containing copy number variation, all Aldy v4.4 calls were concordant with the reference standard except for one *CYP3A4* diplotype call (WES55) in one patient in which WES read depth at the CYP3A4*22 locus was <30. Aldy v4.4 identified additional alleles not covered by the genotyping standard for 385 diplotypes, which included 18 clinically actionable star alleles within *CYP2B6* (2), *CYP2C19* (1), *CYP2D6* (6), *DPYD* (5), *G6PD* (1), *NUDT15* (1), and *SLCO1B1* (2). All additional clinically actionable alleles were orthogonally confirmed during this analysis or during our previous WES validation (17).

**Table 4:** Diplotype-level assessment of Aldy v4.4 genotype extraction from clinical WES.

Gene	Concordant/Equivalent diplotypes^1^	Discordant diplotypes	Detection of additional major star alleles^3,4^	Diplotypes with Copy Number Variation^2^	Indeterminate diplotypes^2^	Total diplotypes compared
*CYP2B6*	96	0	64 (2)	0	(1)	**160**
*CYP2C8*	55	0	2	0	0	**57**
*CYP2C9*	160	0	1	0	0	**161**
*CYP2C19*	142	0	19 (1)	0	0	**161**
*CYP2D6*	101	0	37 (6)	(23)	0	**138**
*CYP3A4*	155	1	5	0	0	**161**
*CYP3A5*	161	0	0	0	0	**161**
*CYP4F2*	118	0	43	0	0	**161**
*DPYD*	43	0	118 (5)	0	0	**161**
*G6PD*	160	0	1(1)	0	0	**161**
*NUDT15*	76	0	3 (1)	0	0	**79**
*SLCO1B1*	72	0	89 (2)	0	0	**161**
*TPMT*	148	0	3	0	(10)	**151**
**Total**	**1487**	1	**385 (18)**	(23)	(11)	**1873**

1.Alleles with the same tag variant(s) (e.g., CYP2D6*2 versus CYP2D6*34 and CYP2D6*39) were considered as equivalent alleles.

2.Since Aldy cannot call copy number changes from whole exome data, all CYP2D6 calls with copy number variation (as called by the genotyping reference standard) were removed from the analysis. These values in parentheses, in addition to subjects with indeterminate diplotypes were not included in diplotype concordance analysis.

3.Additional clinically actionable star alleles were defined as alleles classified as having increased function, decreased function, or no function within current Clinical Pharmacogenetics Implementation Consortium (CPIC) guidelines that were not included on our genotyping reference standard. Since no current CPIC guidelines include *CYP2C8* or *CYP3A4* genotype-guided recommendations, there was no potential to identify additional clinically actionable star alleles. Diplotypes containing additional clinically actionable star alleles are in parentheses.

4.*DPYD* no longer uses star allele nomenclature according to PharmVar, so any additional variant called by Aldy for *DPYD* was interpreted as an additional allele.

As shown in **Table 5**, Aldy v4.4 diplotype calls from WGS data were concordant across all genes except for one *CYP2C9* call (WGS48) and two *CYP2D6* calls (WGS53 and 55). The *CYP2C9* discordant call is puzzling since Aldy v3.3 produced the correct call, and the WGS variant allele frequency was 71% (Aldy v4.4 called the variant homozygous). Notably, two (WGS5 and 35) of the three discordant *CYP2D6* calls from Aldy v3.3 were corrected by v4.4. However, one additional *CYP2D6* discordant call (WGS53) by Aldy v4.4 was identified, which consisted of a copy number discrepancy. Cyrius was run on the four BAMs (WGS5, WGS35, WGS53, and WGS55) with discordant *CYP2D6* Aldy v3.3 or v4.4 (or both calls), generating an accurate genotype call in all four cases (results shown in **Supplemental Table 6**). Aldy v4.4 did correct the *CYP2B6* call (WGS16) that was miscalled by v3.3. Aldy v4.4 identified 232 additional alleles not covered by targeted genotyping in the WGS cohort, including the same 15 clinically actionable alleles that were previously identified by v3.3. Differences between additional major star alleles identified by Aldv v3.3 and v4.4 are due to new updates to Aldy’s gene databases from PharmVar with Aldy v4.4. The intra- and inter-assay concordance of Aldy v4.4 for all assessed variants was confirmed to be 100% using both WES and WGS data. All diplotype calls from Aldy v4.4, Aldy v3.3, and the genotyping reference standard for the WES and WGS cohorts are provided in **Supplemental File 1**.

**Table 5:** Diplotype-level assessment of Aldy v4.4 genotype extraction from clinical WGS.

Gene	Concordant/Equivalent diplotypes^1^	Discordant diplotypes	Detection of additional major star alleles^2,3^	Indeterminate diplotypes^4^	*CYP2D6* Hybrid diplotype calls^4^	Total diplotypes compared
*CYP2B6*	63	0	30 (1)	**(1)**	N/A	**93**
*CYP2C8*	91	0	3	**0**	N/A	**94**
*CYP2C9*	92	1	1	**0**	N/A	**94**
*CYP2C19*	84	0	10 (3)	**0**	N/A	**94**
*CYP2D6*	58	2	26	**0**	(8)	**87**
*CYP3A4*	91	0	3	**0**	N/A	**94**
*CYP3A5*	94	0	0	**0**	N/A	**94**
*CYP4F2*	68	0	26	**0**	N/A	**94**
*DPYD*	21	0	73 (8)	**0**	N/A	**94**
*G6PD*	94	0	0	**0**	N/A	**94**
*NUDT15*	0	0	1 (1)	**0**	N/A	**1**
*SLCO1B1*	38	0	56 (2)	**0**	N/A	**94**
*TPMT*	87	0	3	**(4)**	N/A	**90**
*VKORC1*	94	0	0	**0**	N/A	**94**
**Total**	**975**	**3**	**232 (15)**	**(5)**	**(8)**	**1211**

1.Alleles with the same tag variant(s) (e.g., CYP2D6*2 versus CYP2D6*34 and CYP2D6*39) were considered as equivalent alleles.

2.Additional clinically actionable star alleles were defined as alleles classified as having increased function, decreased function, or no function within current Clinical Pharmacogenetics Implementation Consortium (CPIC) guidelines that were not included on our genotyping reference standard. Since no current CPIC guidelines include *CYP2C8* or *CYP3A4* genotype-guided recommendations, there was no potential to identify additional clinically actionable star alleles. Diplotypes containing additional clinically actionable star alleles are in parentheses.

3.*DPYD* no longer uses star allele nomenclature according to PharmVar, so any additional variant called by Aldy for DPYD was interpreted as an additional allele.

4.Values in parentheses were not included in diplotype concordance analysis for subjects with indeterminate diplotypes or *CYP2D6* hybrid calls.

### Clinical impact of additional star alleles identified by Aldy

Thirty patients (11.5% of the study population), consisting of 13 and 17 patients from the WGS and WES cohorts, respectively, had additional clinically actionable star alleles conclusively identified by Aldy (two subjects [WGS69 and WES33] had additional clinically actionable *CYP2B6* alleles that were unable to be unambiguously phased). Genotype-predicted phenotypes for these patients, as predicted by Aldy results and by results from the genotyping reference standard, are shown in **Table 6**; in addition, **Table 6** summarizes prescriptions for actionable medications (i.e., those with different CPIC recommendations based on Aldy-predicted and genotyping-predicted phenotypes) since each patients’ respective date of first cancer diagnosis. Twenty-one of the 30 patients had medication data available, which included 289 ± 460 (median ± interquartile range) unique prescriptions per patient during the 7.3 ± 7.5 year follow-up period, defined as each patient’s date of first cancer diagnosis until the end of the data collection period. Of these 21 patients, 5 (23.8%) were prescribed a medication which had a different CPIC recommendation based on their Aldy-predicted pharmacogene phenotype.

**Table 6:** Genotype-predicted phenotypes based on genotyping reference standard and Aldy v4.4 results, including whether patients were prescribed actionable medications since their respective dates of first cancer diagnosis.

Patient ID	Follow up (years)*	Gene	Genotyping Panel		Aldy v4.4		Actionable Medications (# of prescriptions)^+^
			Diplotype	Phenotype	Diplotype	Phenotype	
WGS29	13.8	*DPYD*	*1/*1	NM (AS=2.0)	*5+rs1801265/*HapB3: *9/*HapB3+rs1801159	IM (AS=1.5)	Fluorouracil (1)
WGS34	3.1	*DPYD*	*1/*1	NM (AS=2.0)	*9/*HapB3	IM (AS=1.5)	Fluorouracil (20)
WGS41	5.4	*CYP2C19*	*1/*2	IM	*2/*35	PM	None
WGS45	8.8	*DPYD*	*1/*1	NM (AS=2.0)	*1/*7	IM (AS=1.0)	None
WGS53	11.5	*NUDT15*	*1/*1	NM	*1/*9	IM	None
WGS54	2.9	*DPYD*	*1/*1	NM (AS=2.0)	*9+rs2297595/*9+rs115232898	IM (AS=1.5)	Capecitabine (1)
		*SLCO1B1*	*1/*1	NF	*31/*37	DF	
WGS56	4.7	*DPYD*	*1/*1	NM (AS=2.0)	*HapB3/*rs17376848	IM (AS=1.5)	No Data
WGS62	2.9	*CYP2C19*	*1/*1	NM	*1/*9	Likely IM	No Data
WGS73	2.6	*DPYD*	*1/*1	NM (AS=2.0)	*9/*HapB3	IM (AS=1.5)	No Data
WGS77	7.4	*DPYD*	*1/*1	NM (AS=2.0)	*9+rs1801159/*rs67376798	IM (AS=1.5)	None
WGS93	4.9	*DPYD*	*1/*1	NM (AS=2.0)	*rs17376848/*rs67376798	IM (AS=1.5)	None
WGS96	2.7	*SLCO1B1*	*1/*1	NF	*27/*31	Possibly DF	No Data
WGS97	4.2	*CYP2C19*	*1/*1	NM	*1/*35	IM	No Data
WES7	9.4	*CYP2D6*	*1/*4	IM (AS=1.0)	*4.021/*15	PM (AS=0)	Tramadol (3)
WES11	1.4	*DPYD*	*1/*1	NM (AS=2.0)	*9+rs56038477/*rs2297595	IM (AS=1.5)	No Data
WES19	15.0	*DPYD*	*1/*1	NM (AS=2.0)	*5+rs2297595+rs1801265/*rs67376798	IM (AS=1.5)	None
WES23	15.1	*CYP2D6*	*2/*4	IM (AS=1.0)	*4.021/*59	IM (AS=0.5)	None
WES43	12.9	*DPYD*	*1/*1	NM (AS=2.0)	*1+rs56038477/*9	IM (AS=1.5)	Fluorouracil (10)
WES65	3.5	*CYP2C19*	*1/*2	IM	*2/*24	PM	None
WES68	1.0	*G6PD*	A/A	Normal	*A/*G6PDA-968C_376G	Variable	No Data
WES71	3.6	*DPYD*	*1/*1	NM (AS=2.0)	*1/*rs67376798	IM (AS=1.5)	None
WES92	0.9	*NUDT15*	*1/*1	NM	*1/*9	IM	No Data
WES93	3.9	*SLCO1B1*	*1/*1	NF	*31/*37	DF	None
WES101	2.5	*CYP2D6*	*1/*2	NM (AS=2.0)	*1/*59	NM (AS=1.5)	N/A^=^
WES102	2.0	*CYP2D6*	*1/*2	NM (AS=2.0)	*1/*59	NM (AS=1.5)	No Data; N/A^=^
WES108	5.5	*CYP2D6*	*1/*1	NM (AS=2.0)	*1/*15	IM (AS=1.0)	None
WES124	3.0	*DPYD*	*1/*1	NM (AS=2.0)	*1/*rs67376798	IM (AS=1.5)	None
WES128	7.7	*CYP2D6*	*1/*41	NM (AS=1.25)	*41/*62	IM (AS=0.25)	None
WES137	15.7	*CYP2B6*	*1/*9	IM	*8+rs3211371/*9	PM	None
WES154	4.2	*SLCO1B1*	*1/*1	NF	*14/*31	DF	None

AS, activity score; DF, decreased function; IM, intermediate metabolizer; N/A, not applicable; NF, normal function; NM, normal metabolizer; No Data, patient medication data was not available after the date of first cancer diagnosis; PM, poor metabolizer.

*Follow up is presented in years and was defined as the amount of time between the patient’s date of cancer diagnosis and the end of the medication data collection period (10/01/2020).

^+^Medications were defined as “actionable” if current CPIC guidelines provide “strong” or “moderate” recommendations to alter therapy with the medication based on each patient’s Aldy-predicted phenotype (relative to their genotyping-predicted phenotype).

^=^N/A describes situations in which CPIC guidelines provide identical recommendations for all medications based on the patient’s Aldy-predicted and genotyping-predicted phenotypes.

## Discussion

In this investigation, we assessed the performance of the two most recent major versions of the Aldy computational tool (v3.3, released on 07/17/21; v4.4, released on 12/14/22) (26) in extracting PGx genotypes from 255 clinically obtained NGS files for 13-14 major pharmacogenes. Our findings demonstrate that Aldy v3.3 diplotype calls from WGS data were concordant with the genotyping reference standard for 1005 of 1010 calls, yielding an overall accuracy of 99.5%. In a previous investigation, we found that, when excluding genotypes with *CYP2D6* copy number variation, Aldy v3.3 diplotype calls from WES data were perfectly concordant with the genotyping reference standard for 736 diplotypes in the validation cohort (17). When testing Aldy v4.4 on these same NGS files, we observed a diplotype call overall accuracy rate of 975/978 (99.7%) in the WGS cohort and 1487/1488 (99.9%) in the WES cohort. In addition, Aldy v3.3 identified additional star alleles not covered by genotyping for 196 diplotype calls, in which 15 diplotypes contained alleles with actionable CPIC recommendations. Within the same WGS cohort, Aldy v4.4 identified additional star alleles for 232 diplotype calls, including the same 15 diplotypes with actionable CPIC recommendations identified by Aldy v3.3. This study demonstrates the benefits of using sequencing data with Aldy-based genotype extraction to identify additional clinically actionable variants not covered by standard genotyping assays, as demonstrated by our findings that 23.8% of patients with additional actionable alleles were prescribed a medication where CPIC guidelines provide different recommendations. However, it is important to also consider potential challenges when identifying and interpreting additional alleles from sequencing data that may not have clinical relevance or defined function.

Past studies have compared the analytical performance of computational genotype extraction tools, including Aldy, Astrolabe, Cyrius, Stargazer, and StellarPGx (12, 13, 18, 27) Although results vary among these analyses, Aldy, Astrolabe, Cyrius and StellarPGx consistently demonstrate >80% accuracy for genotype extraction from WGS for complex *CYP2D6* reference samples that include full gene deletions, full gene duplications, and hybrid rearrangements containing *CYP2D6* and *CYP2D7*. Recently, Aldy v4.0 was shown to have an accuracy of >98% across all tested short-read sequencing platforms, including an accuracy of >99% for Illumina WGS (18). Our results expand on these findings to demonstrate Aldy v4.4’s ability to produce >99% accuracy when using clinically obtained NGS data. In comparison to most other platforms, Aldy also offers additional functionalities with clinical utility, including efficient adaptation for use with WES data and the ability to genotype non-CYP enzyme genes with CPIC recommendations, including *CFTR*, *DPYD*, *G6PD*, *IFNL3*, *NUDT15*, *SLCO1B1*, *TPMT*, *UGT1A1*, and *VKORC1* (24).

No diagnostic test is perfectly accurate. Based on our experience testing Aldy’s performance using clinical NGS data, we observed that the primary source of inaccuracy stemmed from the inherent variability in NGS data, which included modest but technically significant variability in coverage and read depth at PGx-relevant loci and the rare potential for apparent sequencing errors to influence Aldy calls. An essential consideration before running Aldy is to thoroughly characterize the coverage and read depth of the sequencing input. In our analyses, we found that mean NGS coverage >30x for all assessed PGx variants was sufficient for Aldy to perform accurate genotype determination. When using Aldy on WES data, it is critical to determine whether clinically relevant non-coding variants (e.g., CYP2C19*17) are covered by the platform’s target capture method. Given the non-uniform coverage patterns, Aldy cannot determine *CYP2D6* copy number from WES data, making it unable to perform *CYP2D6* genotyping in accordance with the minimum recommended variants to test by the Association for Molecular Pathology (28). Relative to experimental methods that involve physical reagents prone to degradation or batching imperfections, computational methods offer a theoretical advantage in terms of reproducibility; indeed, the reproducibility of Aldy genotype extraction from WES and WGS was demonstrated to be perfect within our intra- and inter-assay concordance analyses.

Given their efficient workflows, adaptability to multiple genetic data sources, and high accuracy, computational genotyping platforms are emerging as useful tools to enable PGx research (29–32). To our knowledge, these approaches have not yet been widely implemented to support clinical care. When devising strategies to clinically implement Aldy-based NGS genotype extraction at a major academic medical center, we encountered the following two challenges (1): the need to perform orthogonal confirmation for every new clinically actionable variant detected by Aldy and (2) the inability to truly validate Aldy unless the sequencing and bioinformatics workflows are controlled as part of the process. With plans to establish an institutional clinical sequencing laboratory, we ultimately envision clinically validating Aldy to allow us to embed extracted genotypes into the electronic health record to interact with existing clinical decision support modalities (e.g., best practice alerts) and thereby promote genotype-guided prescribing. However, based on this investigation and our previous WES validation (17), we raise a number of considerations to ensure accurate Aldy genotyping when using clinical sequencing data.

Important considerations to ensure accurate Aldy-based genotype extraction from clinical sequencing data:

1. The need to confirm that pharmacogenomic loci of interest are adequately covered (i.e., read depth ≥ 30x), which should include variants defined by the Association for Molecular Pathology as the minimum set to include in PGx genotyping assays for *CYP2C9* (33), *CYP2C19* (34), *CYP2D6* (28), *NUDT15*, *TPMT* (35), and *VKORC1* (36)
2. Aldy cannot phase potentially ambiguous haplotypes (e.g., TPMT *1/*3A vs. *3B/*3C), which require additional testing, such as phased genetic testing or activity assays, to conclusively resolve
3. Aldy will only call variants included in its gene databases (available online at: https://github.com/0xTCG/aldy/tree/master/aldy/resources/genes) and will not identify novel variants
4. Aldy cannot determine *CYP2D6* copy number from WES, which requires additional genetic testing
5. Aldy’s ability to accurately call hybrid alleles containing *CYP2D6* and *CYP2D7* from clinical sequencing data has not been confirmed and requires validation using an appropriate orthogonal platform (e.g., long-read sequencing)

We acknowledge the following limitations of our investigation. For variants not included within the panel-based genotyping platform, orthogonal confirmation methods were only performed for samples identified by Aldy as having the relevant variants. As a result, we were only able to assess the analytical sensitivity of Aldy in detecting these variants and not the analytical specificity. Additionally, our assessments were confined to the variants that were detected in our moderately sized, predominantly White study population. Therefore, clinically actionable pharmacogene variants that are relatively rare or are found in other racial/ethnic groups were not able to be assessed. We also did not confirm Aldy’s performance in calling hybrid alleles containing *CYP2D6* and *CYP2D7* from WGS, which would require extensive characterization using multiple testing platforms including additional sequencing and *CYP2D6* copy number testing. However, consideration of Aldy for clinical *CYP2D6* testing from WGS would require an extensive validation of Aldy’s ability to accurately call complex *CYP2D6* structural variation and confirmation using appropriate reference materials and patient samples. Finally, we did not attempt to assess *UGT1A1*, which is supported by Aldy and included in CPIC recommendations to guide atazanavir therapy (37), because it is not included on our genotyping panel due to atazanavir not being widely prescribed at our institution.

In conclusion, our findings demonstrate Aldy’s ability to extract pharmacogenotypes from clinical WES and WGS data with high accuracy. Future work is needed to further optimize Aldy-based genotyping and determine optimal strategies to implement Aldy-based genotype extraction from clinical NGS data to inform genotype-guided prescribing.

## Data availability statement

The genomic sequencing data presented in this article cannot be deposited into a public database due to ethical approval restrictions on sharing the data publicly. Requests to access the datasets should be directed to TCS (tskaar@iu.edu).

## Ethics statement

The studies involving human participants were reviewed and approved by Indiana University Institutional Review Board. The patients/participants provided their written informed consent to participate in this study.

## Author contributions

TS, RL, WO, ER, CG, TL, EM, and RR analyzed the data. TS, RL, JH, AB, BPS, SS, IN, BAS, SB, RR, and TCS contributed to the conception and design of the study. TS and RL wrote the manuscript. All authors contributed to the article and approved the submitted version.
