# Supplementary material for: Computational pharmacogenotype extraction from clinical next-generation sequencing
Source: Front Oncol. 2023 Jul 4;13:1199741. doi: 10.3389/fonc.2023.1199741 (PMC10352904; doi:10.3389/fonc.2023.1199741)
Supplement: Supplementary file 2 [file Table_1.docx]

**Supplemental Table 1. Details of variants included on panel-based genotyping reference standard.**

| **Gene** | **Allele** | **Variant** | **Minor Allele Frequency^+^** | | | **Functional Effect*** |
| --- | --- | --- | --- | --- | --- | --- |
|  |  |  | **African** | **American Admixed** | **European** |  |
| ***CYP2B6*** | *6 | rs3745274 | 0.32 | -- | 0.23 | Decreased |
|  | *18 | rs28399499 | 0.03 | -- | 0.00 | No Function |
| ***CYP2C8*** | *2 | rs11572103 | 0.07 | -- | 0.00 | Likely Decreased |
|  | *3 | rs11572080 | 0.02 | -- | 0.10 | Likely Decreased |
|  | *4 | rs1058930 | 0.01 | -- | 0.04 | Likely Decreased |
| ***CYP2C9*** | *2 | rs1799853 | 0.02 | 0.03 | 0.13 | Decreased |
|  | *3 | rs1057910 | 0.01 | 0.03 | 0.08 | No Function |
|  | *5 | rs28371686 | 0.01 | 0.00 | 0.00 | Decreased |
|  | *6 | rs9332131 | 0.01 | 0.00 | 0.00 | No Function |
|  | *8 | rs7900194 | 0.06 | 0.02 | 0.00 | Decreased |
|  | *11 | rs28371685 | 0.01 | 0.00 | 0.00 | Decreased |
| ***CYP2C19*** | *2 | rs4244285 | 0.18 | 0.12 | 0.15 | No Function |
|  | *3 | rs4986893 | 0.00 | 0.00 | 0.00 | No Function |
|  | *4 | rs28399504 | 0.00 | 0.00 | 0.00 | No Function |
|  | *6 | rs72552267 | 0.00 | 0.00 | 0.00 | No Function |
|  | *8 | rs41291556 | 0.00 | 0.00 | 0.00 | No Function |
|  | *10 | rs6413438 | 0.00 | -- | 0.00 | Decreased |
|  | *17 | rs12248560 | 0.21 | 0.09 | 0.22 | Increased |
| ***CYP2D6*** | *2 | rs16947; rs1135840 | 0.16 | 0.22 | 0.28 | Normal |
|  | *3 | rs35742686 | 0.00 | 0.00 | 0.02 | No Function |
|  | *4 | rs3892097 | 0.05 | 0.10 | 0.19 | No Function |
|  | *5 | Gene deletion | 0.05 | 0.02 | 0.03 | No Function |
|  | *6 | rs5030655 | 0.00 | 0.00 | 0.01 | No Function |
|  | *7 | rs5030867 | 0.00 | 0.01 | 0.00 | No Function |
|  | *8 | rs5030865 | 0.00 | 0.00 | 0.00 | No Function |
|  | *9 | rs5030656 | 0.00 | 0.00 | 0.03 | Decreased |
|  | *10 | rs1065852; rs1135840 | 0.04 | 0.01 | 0.02 | Decreased |
|  | *14 | rs5030865 | 0.00 | 0.00 | 0.00 | Decreased |
|  | *17 | rs28371706 | 0.17 | 0.00 | 0.00 | Decreased |
|  | *29 | rs59421388 | 0.09 | 0.00 | 0.00 | Decreased |
|  | *41 | rs28371725 | 0.04 | 0.02 | 0.09 | Decreased |
| ***CYP3A4*** | *2 | rs55785340 | 0.00 | -- | 0.00 | Decreased |
|  | *22 | rs35599367 | 0.00 | -- | 0.05 | Decreased |
| ***CYP3A5*** | *3 | rs776746 | 0.32 | -- | 0.92 | No Function |
|  | *6 | rs10264272 | 0.11 | -- | 0.00 | No Function |
|  | *7 | rs41303343 | 0.12 | -- | 0.00 | No Function |
| ***CYP4F2*** | *3 | rs2108622 | 0.08 | 0.41 | 0.30 | Decreased |
| ***DPYD*** | *2 | rs3918290 | 0.00 | -- | 0.01 | No Function |
| ***G6PD*** | A | rs1050829 | 0.27 | -- | 0.00 | Normal |
|  | A- | rs1050828 | 0.11 | -- | 0.00 | Deficient |
| ***SLCO1B1*** | *5 | rs4149056 | 0.00 | -- | 0.02 | Decreased |
| ***TPMT*** | *2 | rs1800462 | 0.01 |  | 0.00 | No Function |
|  | *3 | rs1800460; rs1142345 | 0.03 | -- | 0.03 | No Function |
| ***VKORC1*** | -1639G>A | rs9923231 | 0.11 | 0.62 | 0.41 | Increased Warfarin Sensitivity |

**Key**: **--** = Data not available

+Population-specific minor allele frequencies were reported based on information from PharmGKB’s “PGx Gene-specific Information Tables” (<https://www.pharmgkb.org/page/pgxGeneRef>, last accessed 01/13/23).

*Functional effects for each variant are defined by the Pharmacogene Variation Consortium (<http://pharmvar.org>, last accessed: 01/13/23), Clinical Pharmacogenetics Implementation Consortium (<http://cpicpgx.org>, last accessed: 01/13/23), or the Human Cytochrome P450 (CYP) Allele Nomenclature Committee (originally available at: cypalleles.ki.se; now archived at: <https://www.pharmvar.org/htdocs/archive/index_original.htm>, last accessed: 01/13/23).

**Supplemental Table 2. Assay identification numbers for TaqMan reagents and sequences of Sanger sequencing primers used for orthogonal confirmation.**

| **Gene** | **Allele** | **Variant** | **TaqMan Assay Identification Number**  **or Sanger Primer Sequence (5’ to 3’)*** |
| --- | --- | --- | --- |
|  |  |  |  |
| ***CYP2B6*** | *8,*13 | rs12721655 | C__30634236_20 |
| ***CYP2C19*** | *9 | rs17884712 | C__25745302_30 |
|  | *2,*35 | rs12769205 | C__25744790_10 |
| ***DPYD*** | *7 | rs72549309 | Forward: **TGTAAAACGACGGCCAGT**ACTAGACACGGACTCTGAATGAG |
|  |  |  | Reverse: **CAGGAAACAGCTATGACC**TGGATTTGCTAAGACAAGCTG |
|  | D949V | rs67376798 | C__27530948_10 |
|  | HapB3 | rs75017182 | C_104846637_10 |
|  | Y186C | rs115232898 | C_165900856_10 |
| ***SLCO1B1*** | *9,*31 | rs59502379 | C__30633910_10 |
| ***NUDT15*** | *9 | rs746071566 | Forward: **TGTAAAACGACGGCCAGT**CATTCCCCAACCTGATAGCC |
|  |  |  | Reverse: **CAGGAAACAGCTATGACC**CAACCGAGCCTTTCCTCTTC |

*Bold nucleotides indicate the M13 tail

**Supplemental Table 3. Medications that were considered “actionable” based on patients’ Aldy v4.4-predicted phenotypes relative to their genotyping-predicted phenotypes, defined as those with different CPIC recommendations.**

| **Patient ID** | **Gene** | **Genotyping-Predicted Phenotype** | **Aldy v4.4- Predicted Phenotype** | **Medications with Different CPIC Recommendations Based on Genotyping- and Aldy v4.4-Predicted Phenotypes** |
| --- | --- | --- | --- | --- |
| WGS29 | *DPYD* | NM (AS=2.0) | IM (AS=1.5) | Capecitabine, Fluorouracil |
| WGS34 | *DPYD* | NM (AS=2.0) | IM (AS=1.5) | Capecitabine, Fluorouracil |
| WGS41 | *CYP2C19* | IM | PM | Amitriptyline, Clomipramine, Doxepin, Imipramine, Trimipramine, Citalopram, Escitalopram, Sertraline, Voriconazole, Omeprazole, Lansoprazole, Pantoprazole* |
| WGS45 | *DPYD* | NM (AS=2.0) | IM (AS=1.0) | Capecitabine, Fluorouracil |
| WGS53 | *NUDT15* | NM | IM | Azathioprine, Mercaptopurine, Thioguanine |
| WGS54 | *DPYD* | NM (AS=2.0) | IM (AS=1.5) | Capecitabine, Fluorouracil, Atorvastatin, Lovastatin, Pitavastatin, Simvastatin |
|  | *SLCO1B1* | NF | DF |  |
| WGS56 | *DPYD* | NM (AS=2.0) | IM (AS=1.5) | Capecitabine, Fluorouracil |
| WGS62 | *CYP2C19* | NM | Likely IM | Clopidogrel, Citalopram, Escitalopram, Sertraline |
| WGS73 | *DPYD* | NM (AS=2.0) | IM (AS=1.5) | Capecitabine, Fluorouracil |
| WGS77 | *DPYD* | NM (AS=2.0) | IM (AS=1.5) | Capecitabine, Fluorouracil |
| WGS93 | *DPYD* | NM (AS=2.0) | IM (AS=1.5) | Capecitabine, Fluorouracil |
| WGS96 | *SLCO1B1* | NF | Possibly DF | Atorvastatin, Lovastatin, Pitavastatin, Simvastatin |
| WGS97 | *CYP2C19* | NM | IM | Clopidogrel, Citalopram, Escitalopram, Sertraline |
| WES7 | *CYP2D6* | IM (AS=1.0) | PM (AS=0) | Amitriptyline, Nortriptyline, Atomoxetine, Codeine, Tramadol, Paroxetine, Fluvoxamine, Vortioxetine, Tamoxifen |
| WES11 | *DPYD* | NM (AS=2.0) | IM (AS=1.5) | Capecitabine, Fluorouracil |
| WES19 | *DPYD* | NM (AS=2.0) | IM (AS=1.5) | Capecitabine, Fluorouracil |
| WES23 | *CYP2D6* | IM (AS=1.0) | IM (AS=0.5) | Atomoxetine, Tamoxifen^+^ |
| WES43 | *DPYD* | NM (AS=2.0) | IM (AS=1.5) | Capecitabine, Fluorouracil |
| WES65 | *CYP2C19* | IM | PM | Amitriptyline, Clomipramine, Doxepin, Imipramine, Trimipramine, Citalopram, Escitalopram, Sertraline, Voriconazole, Omeprazole, Lansoprazole, Pantoprazole* |
| WES68 | *G6PD* | Normal | Variable | Dapsone, Methylene blue, Pegloticase, Primaquine, Rasburicase, Tafenoquine, Toluidine blue |
| WES71 | *DPYD* | NM (AS=2.0) | IM (AS=1.5) | Capecitabine, Fluorouracil |
| WES92 | *NUDT15* | NM | IM | Azathioprine, Mercaptopurine, Thioguanine |
| WES93 | *SLCO1B1* | NF | DF | Atorvastatin, Lovastatin, Pitavastatin, Simvastatin |
| WES101 | *CYP2D6* | NM (AS=2.0) | NM (AS=1.5) | N/A^=^ |
| WES102 | *CYP2D6* | NM (AS=2.0) | NM (AS=1.5) | N/A^=^ |
| WES108 | *CYP2D6* | NM (AS=2.0) | IM (AS=1.0) | Amitriptyline, Nortriptyline |
| WES124 | *DPYD* | NM (AS=2.0) | IM (AS=1.5) | Capecitabine, Fluorouracil |
| WES128 | *CYP2D6* | NM (AS=1.25) | IM (AS=0.25) | Amitriptyline, Nortriptyline, Atomoxetine, Tamoxifen |
| WES137 | *CYP2B6* | IM | PM | Efavirenz |
| WES154 | *SLCO1B1* | NF | DF | Atorvastatin, Lovastatin, Pitavastatin, Simvastatin |

**Abbreviations:** AS = activity score; DF = decreased function; IM = intermediate metabolizer; N/A = not applicable; NF = normal function; NM = normal metabolizer; No Data = patient medication data was not available after the date of first cancer diagnosis; PM = poor metabolizer

*Proton pump inhibitors were considered actionable for a *CYP2C19* genotype-predicted phenotype change from IM to PM since the CPIC guideline recommendation to consider 50% dose reduction goes from “optional” to “moderate.”

^+^Tamoxifen was considered actionable for a *CYP2D6* genotype-predicted phenotype change from AS=1.0 to AS=0.5 since the CPIC guideline recommendation to consider alternative therapy goes from “optional” to “moderate.”

^=^N/A describes situations in which CPIC guidelines provide identical recommendations for all medications based on the patient’s Aldy-predicted and genotyping-predicted phenotypes.

**Supplementary Table 4. Demographic and clinical characteristics of the WGS cohort.**

| **Variable** | **WGS Cohort**  **(n=100)** | | |
| --- | --- | --- | --- |
| Age in years at first cancer diagnosis (Median [IQR]) | 56 (17) | | |
| *Sex (Count [Percent])* |  | | |
| Male | 56 (56.0%) | | |
| Female | 44 (44.0%) | | |
| *Race (Count [Percent])* |  | | |
| White | 85 (85.0%) | | |
| Black | 12 (12.0%) | | |
| Unknown | 3 (3.0%) | | |
| Asian | 0 (0%) | | |
| *Ethnicity (Count [Percent])* |  | | |
| Non-Hispanic | 100 (100%) | | |
| Hispanic | 0 (0%) | | |
| *Primary Cancer type (Count [Percent])* |  | | |
| Breast | 18 (18.0%) | | |
| Colorectal | 8 (8.0%) | | |
| Pancreatic | 10 (10.0%) | | |
| Prostate | 11 (11.0%) | | |
| Soft tissue sarcoma | 4 (4.0%) | | |
| Ovarian | | 5 (5.0%) |  |
| Bladder | | 8 (8.0%) |  |
| Esophageal | | 1 (1.0%) |  |
| Cholangiocarcinoma | | 3 (3.0%) |  |
| Head and neck | | 4 (4.0%) |  |
| Non-small cell lung | | 4 (4.0%) |  |
| Glioblastoma | | 1 (1.0%) |  |
| Melanoma | | 2 (2.0%) |  |
| Renal | | 1 (1.0%) |  |
| Other* | | 20 (20.0%) |  |

*The “other” category consisted of all primary cancer types which occurred in fewer than 5 patients in our full study population.

**Supplemental Table 5. Read depth of the 45 star allele-defining variants included on our genotyping panel reference standard within our patient cohort with whole genome sequencing (n=100).**

| **Variant** | **Average Read Depth (Mean ± SD)** | **Number of Patients with Read Depth <30x** |
| --- | --- | --- |
| *CYP2B6*6* (c.516G>T) | 36 ± 11 | 36 |
| *CYP2B6*18* (c.983T>C) | 34 ± 11 | 38 |
| *CYP2C8*2* (c.805A>T) | 40 ± 10 | 12 |
| *CYP2C8*3* (c.416G>A) | 40 ± 12 | 15 |
| *CYP2C8*4* (c.792C>G) | 40 ± 10 | 13 |
| *CYP2C9*2* (c.430C>T) | 38 ± 11 | 20 |
| *CYP2C9*3* (c.1075A>C) | 37 ± 9 | 17 |
| *CYP2C9*5* (c.1080C>G) | 37 ± 9 | 18 |
| *CYP2C9*6* (c.818delA) | 41 ± 10 | 9 |
| *CYP2C9*8* (c.449G>A) | 39 ± 11 | 13 |
| *CYP2C9*11* (c.1003C>T) | 38 ± 9 | 24 |
| *CYP2C19*2* (c.681G>A) | 40 ± 9 | 8 |
| *CYP2C19*3* (c.636G>A) | 40 ± 9 | 8 |
| *CYP2C19*4* (c.1A>G) | 40 ± 10 | 13 |
| *CYP2C19*6* (c.395G>A) | 38 ± 11 | 20 |
| *CYP2C19*8* (c.358T>C) | 37 ± 11 | 25 |
| *CYP2C19*10* (c.680C>T) | 40 ± 9 | 8 |
| *CYP2C19*17* (g.-806C>T) | 40 ± 10 | 11 |
| *CYP2D6*2* (c.886C>T) | 39 ± 14 | 22 |
| *CYP2D6*2* (c.1457G>C) | 36 ± 15 | 39 |
| *CYP2D6*3* (c.775del) | 35 ± 12 | 35 |
| *CYP2D6*4* (c.506-1G>A) | 40 ± 16 | 24 |
| *CYP2D6*6* (c.454del) | 42 ± 16 | 15 |
| *CYP2D6*7* (c.971A>C) | 37 ± 14 | 27 |
| *CYP2D6*8* (c.505G>T) | 41 ± 17 | 27 |
| *CYP2D6*9* (c.841_843del) | 33 ± 13 | 48 |
| *CYP2D6*10* (c.100C>T) | 36 ± 16 | 45 |
| *CYP2D6*14* (c.505G>A) | 41 ± 17 | 27 |
| *CYP2D6*17* (c.320C>T) | 40 ± 16 | 22 |
| *CYP2D6*29* (c.1012G>A) | 45 ± 18 | 13 |
| *CYP2D6*41* (c.985+39G>A) | 37 ± 14 | 29 |
| *CYP3A4*2* (c.664T>C) | 39 ± 10 | 17 |
| *CYP3A4*22* (c.522-191C>T) | 37 ± 9 | 25 |
| *CYP3A5*3* (c.219-237A>G) | 40 ± 11 | 17 |
| *CYP3A5*6* (c.624G>A) | 38 ± 10 | 16 |
| *CYP3A5*7* (c.1035dup) | 42 ± 11 | 11 |
| *CYP4F2*3* (c.1297G>A) | 36 ± 12 | 34 |
| *DPYD*2* (c.1905+1G>A) | 40 ± 10 | 7 |
| *G6PD* A- (c.202G>A) | 29 ± 15 | 57 |
| *G6PD* A (c.376A>G) | 29 ± 15 | 57 |
| *SLCO1B1*5* (c.521T>C) | 43 ± 10 | 5 |
| *TPMT*2* (c.238G>C) | 38 ± 9 | 14 |
| *TPMT*3* (c.460G>A) | 41 ± 11 | 12 |
| *TPMT*3* (c.719A>G) | 40 ± 10 | 10 |
| *VKORC1* (c.-1639G>A) | 36 ± 10 | 24 |

**Supplemental Table 6. Comparison of Aldy v3.3 and v4.4 and Cyrius v1.1.1 *CYP2D6* genotype calls for five subjects with Aldy calls that were discordant with genotyping reference standard.**

| **Patient ID** | **Aldy v3.3** | | **Aldy v4.4** | | **Cyrius v1.1.1** | | **Reference Standard** |
| --- | --- | --- | --- | --- | --- | --- | --- |
|  | **Genotype Call** | **Concordant with Reference?** | **Genotype Call** | **Concordant with Reference?** | **Genotype Call** | **Concordant with Reference?** | **Genotype Call** |
| WGS5 | *1/*39 | **No** | *1/*2 | Yes | *1/*2 | Yes | *1/*2 |
| WGS35 | *1+rs769258/*34 | **No** | *1/*35 | Yes | *1/*35 | Yes | *1/*2 |
| WGS53 | *1+*1/*68+*4.021 | Yes | *4C/*139 | **No** | *1x2/*68+*4 | Yes | *1/*4/xN |
| WGS55 | *34/*35 | **No** | *34/*35 | **No** | *1/*35 | Yes | *1/*2 |
